# To Filter Water

**Published:** 1875-01

**Authors:** 


To Filter Water.—If any one desires pure water and is in doubt whether his water-supply furnishes it, he must filter it. It is fair to say that nearly all the water drank contains animal, vegetable or mineral additions It is a remarkable fact, in view of the great noise which vegetarians have made for years against the use of mineral medicaments, that mineral substances infused in drinking water do far less harm than vegetable impurities. Organic vegetable substances in our drinking water poison it, as well as the blood of the drinker, while water impregnated with certain minerals, speedily acquires a great reputation as a curative agent. In no way can we purify water as surely and as perfectly as by filtering it through the proper mineral substances. The best filter is made from iron and limestone. Iron ore must be used, and any person having such ore at hand can make their own filter, and the best one known to science. The ore must be subjected to heat enough to drive off the sulphur, copper and other volatile matter, and must then be bruised to a coarse powder. The filter is prepared by putting three or four inches of bruised limestone in the bottom of any earthen receptacle having a faucet at the bottom, and placing a few inches of the pounded iron ore on the pounded stone. This removes all albuminoid and nitrogenized compounds, and also lead-contaminations from the water, and a trace of iron taken up by the water is separated by its subsequent passage through the limestone. One charge of the material thus described, and costing nothing but a little trouble, secures efficient filtering for ten gallons of water per day during 200 days.

---

Oat Meal.—It is a bad practice to eat by square and compass, and conclusive proof of a want of force of character, to have the thoughts dwell upon what shall be on the table at the next meal. Yet there are certain general principles connected with the food we eat, which intelligent persons should strictly bear in mind.

The Scotch, more than the Belgians, and French, and English, are strong in the arms, and chest and loins. For the greater part of their youth, Scotch children make at least one entire meal each day of oat meal porridge—which is a thick mush—and milk ; the same fact was also observed among the Scotch and Scotch-Irish from Ulster, who subsist largely in youth and in mature age on the same kind of food. The resulting bodily power was discovered uniformly, during a series of observations carefully made for a period of twenty successive years. Very likely there were elements of climate, character and employment which contributed to the result ; at the same time, it is fair to infer, that the ailment was a controlling cause of bodily vigor.

Oat meal is far richer in elements which make strong bones and hard muscles, than wheat bread, and is not much less nutritious, pound for pound, than roast beef.

Its universal use would make an incalculable improvement in the health of our people, give firmer flesh, stronger bones, and more enduring teeth, because oat meal supplies the needed elements in great abundance. If children, from weaning up to twelve years of age, were required to make their breakfast and supper wholly of oat meal mush and new milk, allowing them their choice of food at the noonday meal, the next generation would be far stronger and better. Sedentary persons, especially the dyspeptic and the billicus, might cure themselves, in many cases, by the same diet, adding to breakfast and supper a cup of hot drink, and half a pound or more of ripe, raw fruits and melons.

